# Anatomical aspects of the insula, opercula and peri-insular white matter for a transcortical approach to insular glioma resection

**DOI:** 10.1007/s10143-021-01602-5

**Published:** 2021-07-22

**Authors:** Tomasz Andrzej Dziedzic, Aleksandra Bala, Andrzej Marchel

**Affiliations:** 1 Department of Neurosurgery, Medical University of Warsaw, Banacha 1a, 02-097 Warszawa, Poland; 2 Faculty of Psychology, University of Warsaw, Warsaw, Poland

**Keywords:** Insula, White matter, Anatomy, Fiber dissection, Tractography, Glioma

## Abstract

The insula is a lobe located deep in each hemisphere of the brain and is surrounded by eloquent cortical, white matter, and basal ganglia structures. The aim of this study was to provide an anatomical description of the insula and white matter tracts related to surgical treatment of gliomas through a transcortical approach. The study also discusses surgical implications in terms of intraoperative brain mapping. Five adult brains were prepared according to the Klingler technique. Cortical anatomy was evaluated with the naked eye, whereas white matter dissection was performed with the use of a microscope. The widest exposure of the insular surface was noted through the temporal operculum, mainly in zones III and IV according to the Berger-Sanai classification. By going through the pars triangularis in all cases, the anterior insular point and most of zone I were exposed. The narrowest and deepest operating field was observed by going through the parietal operculum. This method provided a suitable approach to zone II, where the corticospinal tract is not covered by the basal ganglia and is exposed just under the superior limiting sulcus. At the subcortical level, the identification of the inferior frontoocipital fasciculus at the level of the limen insulae is critical in terms of preserving the lenticulostriate arteries. Detailed knowledge of the anatomy of the insula and subcortical white matter that is exposed through each operculum is essential in preoperative planning as well as in the intraoperative decision-making process in terms of intraoperative brain mapping.

## Introduction

**Fig. 1:**
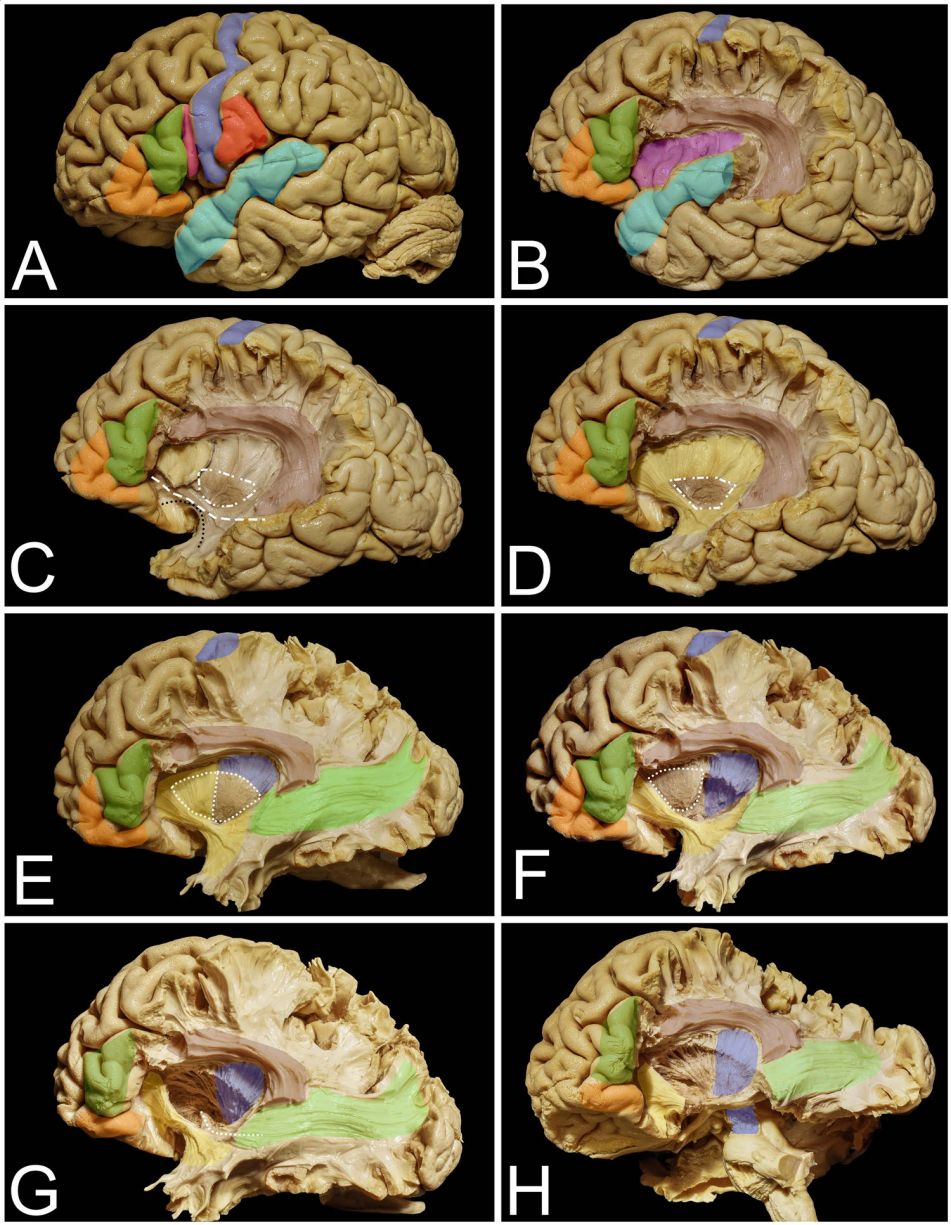
The anatomy of cortical and subcortical structures around and on the mesial surface of the insula. **A** The figure represents the eloquent cortical opercula that surround the insula. Within the IFG, the pars orbitalis (orange), pars triangularis (green), and pars opercularis (pink) within the dominant hemisphere are related mainly to language function. The posterior precentral gyrus (blue) and postcentral gyrus (red) represent the primary motor and sensory cortex, respectively. The superior temporal gyrus (light blue) represents the temporal operculum, and its posterior part represents the so-called Wernicke’s speech area. **B** Resection of the posterior part of the frontal operculum and the parietal operculum exposes the AF/SLF (light burgundy) complex, which is located around the insula in most superficial association fiber systems. In addition, the insular cortex (purple) is exposed. **C** Resection of the insular cortex exposes the extreme capsule (light yellow), which is separated by the claustrum (white square-rectangle line) from the external capsule, which in its anterior and inferior aspect within the limen insulae is composed of UF (black dots) and IFOF (white rectangles) fibers. **D** Resection of the extreme capsule provides exposure of the external capsule (darker yellow), and the claustrum was left in place. **E** With resection of the external capsule and claustrum, the putamen (white dots) is exposed (though it is still anteriorly covered by the external capsule); the corona radiata is located above it and mesial to the AF/SLF complex, representing the corticospinal fibers (blue). Extending resection posteriorly beyond the AF/SLF complex reveals the fibers of the sagittal stratum (light green). **F** Resection of the posterior half of the putamen exposes the fibers of the posterior limb of the internal capsule, which are located deeper than the corona radiata, on the mesial border. **G** Resection of the anterior half of the putamen exposes the anterior perforated substances from the superior region, and the anterior commissure at depth is visible (white squares). **H** Resection of the temporal lobe reveals the corticospinal fibers within the posterior limb of the internal capsule as well as the fibers within the cerebral peduncle

**Fig. 2:**
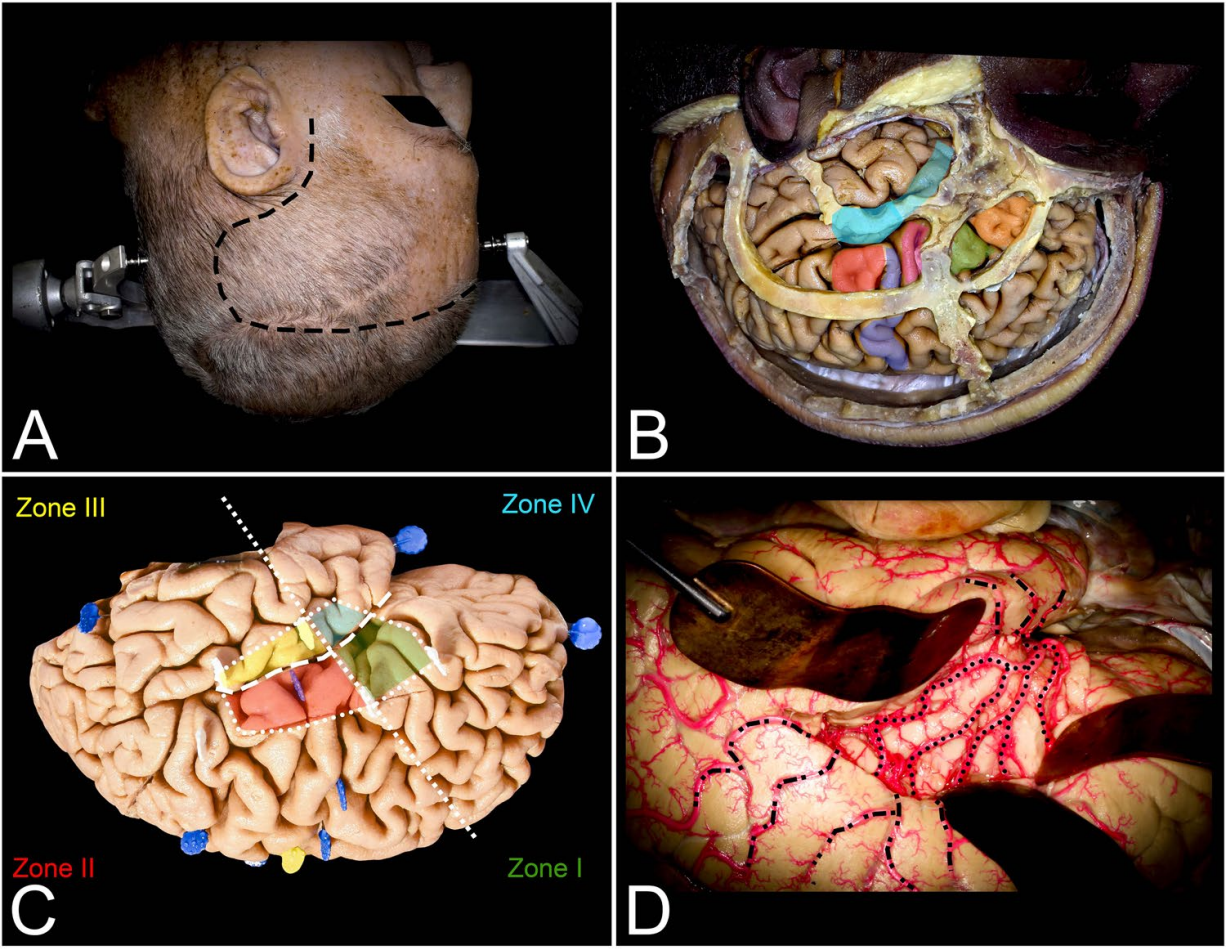
This figure presents the surgical perspective of the insula. **A** The head for the surgical procedure is rotated maximally lateral so that the long axis of the head is nearly parallel to the floor. Depending on the tumor’s localization in relation to the lateral fissure, the head should be tilted inferiorly or superiorly by approximately 15 degrees. To expose all opercula, a curved skin incision (black rectangles) was made posteriorly behind the ear to expose the distal end of the lateral fissure within the inferior parietal lobule. **B** The pars opercularis (pink) of the IFG is located just behind the coronal suture and above the squamosal suture on the dominant side. This region corresponds to Broca’s area. Areas anterior to Broca’s area, anterior to the coronal suture pars triangularis (green) and further anterior to the pars orbitalis (orange), and areas below the superior temporal line were identified. The temporal operculum, which corresponds to the STG, is localized in proximity to the squamosal suture, which approximately follows the lateral sulcus intracranially. The limited (light blue) anterior resection of the STG ends at the level of the sulcus acusticus, whereas the extended resection also involves Heschl’s gyri posteriorly. The parietal operculum (red) involves the inferior segment of the postcentral gyrus, which is located behind the inferior Rolandic point, anterior to the postcentral sulcus and behind the precentral gyrus (blue) and pars opercularis (pink). **C** This representation of the insula (white dots) projected on the lateral surface of the hemisphere allows us to plan the surgical cortical approaches in relation to tumor localization. The most practical technique is to relate tumor localization to two perpendicular lines. The first one follows the lateral sulcus (white rectangles), whereas the other one (white squares) is perpendicular to the previous sulcus and goes through the foramen of Monro. Based on this, the insula is divided into four compartments. The compartments are numbered in clockwise fashion starting from the anterior–superior compartment as follows: zones I (green), II (red), III (yellow), and IV (light blue). **D** The lateral fissure of the left hemisphere was split and opened widely, and the insular cortex was exposed. On its surface, the M2 (black dots) segment of the MCA, which supplies the insula (short perforators) and corona radiata (long perforators), is exposed. Above the insula, on the lateral surface of the hemispheres, the M3, M4, and M5 (black square-rectangular line) segments supplying the lateral surface of the hemisphere are also presented

The insula is the hidden lobe in the hemispheres of the brain and is not seen on the lateral surface of the brain without lateral fissure splitting and opercula retraction [41, 53] (Figs. 1 and 2). The opercula, white matter tracts and basal ganglia around and at the mesial border of the insula are highly eloquent, especially in the dominant hemisphere (Figs. 1 and 2). Additional limitations of exposure are related to the venous system within the lateral sulcus as well as to the middle cerebral artery (MCA) and its branches at the depth of the lateral sulcus [22, 55]. This makes surgical treatment of insular lesions challenging despite novel diagnostic and intraoperative technologies. The transsylvian (TS) and transcortical (TC) corridors are the two main surgical approaches to the insula [8, 39]. The TS approach provides a shorter operative distance and preserves the noninvolved opercular cortex but requires one to work between branches of the MCA, which carries a risk of symptomatic vasospasm and infraction [57, 58]. Additionally, especially on large volume tumors with large posterior extension where wide opening of the lateral fissure and retraction of the opercula is required, a high risk of venous infarction is taken into consideration [3]. The TC approach seems to be a natural choice when the tumor involves one or more opercula, and resection of the superficial part of the tumor provides simultaneous access to the insula, which is present in approximately 77% of insular gliomas [8]. Due to the subpial technique that is used in the TC approach, vessels remain covered by the arachnoid, which reduces the risk of symptomatic vasospasm compared to the TS approach. On the other hand, the TC approach, especially on the dominant side, requires surgery to be performed while the patient is awake as intraoperative cortical and subcortical brain mapping with neuropsychological assessment, including language, must be performed to identify silent brain regions. Intraoperative neuronavigational systems are helpful in cases of insular lesions, but for proper usage and planning of the surgery, an anatomical and functional background of the white matter tracts and opercular cortex is mandatory. The aim of this study was to present the surgical exposure of the insula and surrounding white matter tracts related to insula surgery through the TC approach with morphometric analysis of the approaches in relation to the reproducible anatomical points. This study also discussed the strategy for intraoperative mapping at the cortical and subcortical levels.

## Material and methods

**Fig. 3:**
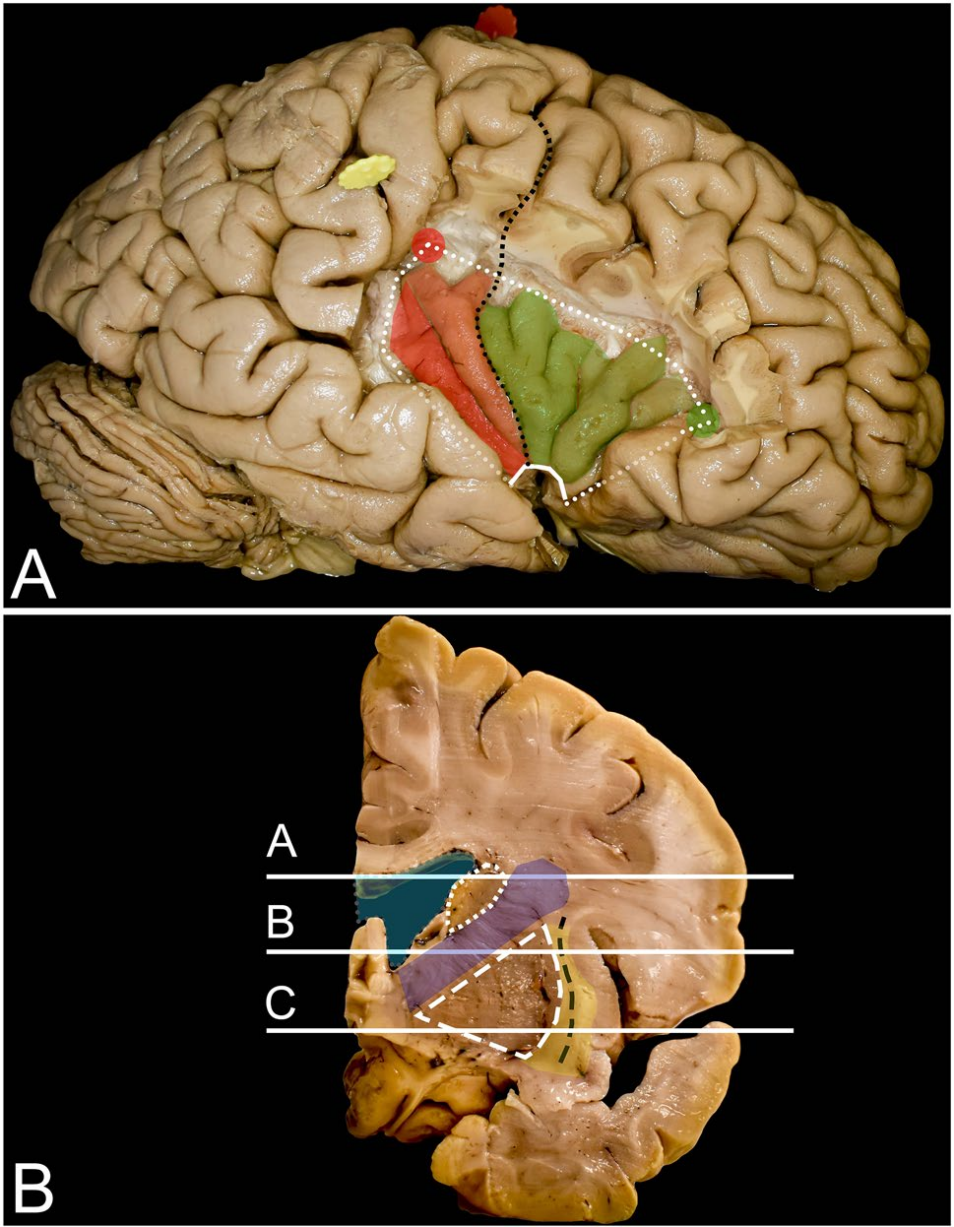
Anatomy of the insula and surrounding structures from the lateral perspective and on coronal brain slices. **A** The insula is enclosed by the limiting sulcus (white dots), which has three rami: the anterior, superior, and inferior ramus; these rami demarcate it from the frontal, frontoparietal and temporal cortex, respectively. The anterior and superior limiting sulci join within the point called the anterior insular point (AIP) (green dot), whereas the superior and inferior sulci join within the posterior insular point (PIP) (red dot). Between the anterior and inferior limiting sulcus, on the ventral surface, the limen insula facing the lesser wing of the sphenoid bone is identified (continuous white line). The most superficial point of the insula is called the insular apex, which is seen mostly within the anterior sylvian point after the arachnoid and vessel removal without any opercular resection or retraction. The insular cortex is composed of three anteriorly placed short (anterior—light green, middle—darker green, and posterior—the darkest green) gyri (anterior part), marked in green, and two posteriorly placed long (anterior—light red) and posterior—darker red) gyri (posterior part of the insula), marked in red. These gyri are separated by the central sulcus of the insula (black dots), which runs from the limen insulae up to the superior limiting sulcus and is in continuation with the central sulcus of the hemisphere (black squares). In some cases, within the anterior compartment of the insula accessory, short gyri are located anteriorly to previously described gyri. **B** Under the insular cortex, an extreme capsule (yellow) is identified, which is separated by the claustrum (black rectangle) from the external capsule (yellow). Mesial to it, the lentiform nucleus (white rectangle) composed of the putamen laterally and globus pallidus medially is identified, which indicates the mesial border of resection. Anterior to the central sulcus, the anterior and posterior ramuses of the internal capsule (blue) are identified medial to the lentiform nucleus and lateral to the lateral ventricle (light blue), and the head of the caudate nucleus is identified superiorly (white squares). Lines a, b, and c correspond to the axial sections of the brain presented in Fig. 4 A, B, and C, respectively

**Fig. 4:**
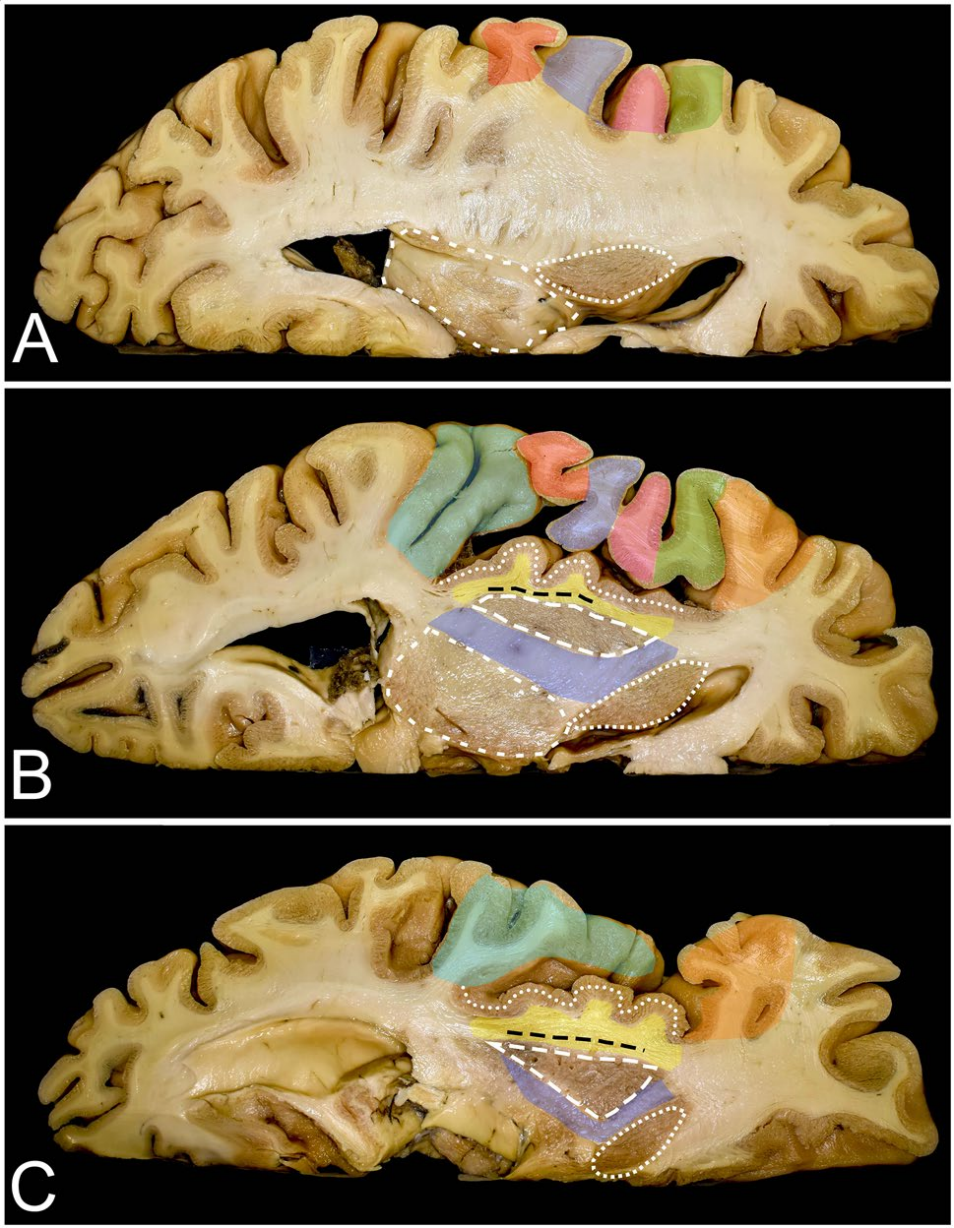
Anatomy of the insula on axial brain slices. **A**, **B**, and **C** correspond to lines a, b, and c presented in the coronal sections in Fig. 3. A, B, and C. The insular cortex is covered by the pars orbitalis (orange), pars triangularis (green), pars opercularis (pink), precentral gyrus (blue), postcentral gyrus (red), and temporal operculum (light blue). After removal of the insular cortex (white dots), the extreme capsule (yellow) separated by the claustrum (black rectangle) from the external capsule (yellow) underneath is exposed. This white matter is formed by the IFOF/UF complex, which is most compacted around the limen insulae. Medial to the external capsule, the lentiform nucleus (white rectangle) is identified. It covers the internal capsule (blue) where corticospinal tract fibers are identified within the posterior limb. The posterior limb of the internal capsule separates the lentiform nucleus from the thalamus (short white lines). Anteriorly, the lentiform nucleus is separated by the anterior ramus of the internal capsule from the head of the caudate nucleus (white rectangle). Above the putamen, the fibers of the internal capsule are part of the corona radiata, and a short segment of these fibers is visible from a lateral perspective when the pars triangularis and opercularis of the IFG as well as parietal opercula are removed

**Fig. 5:**
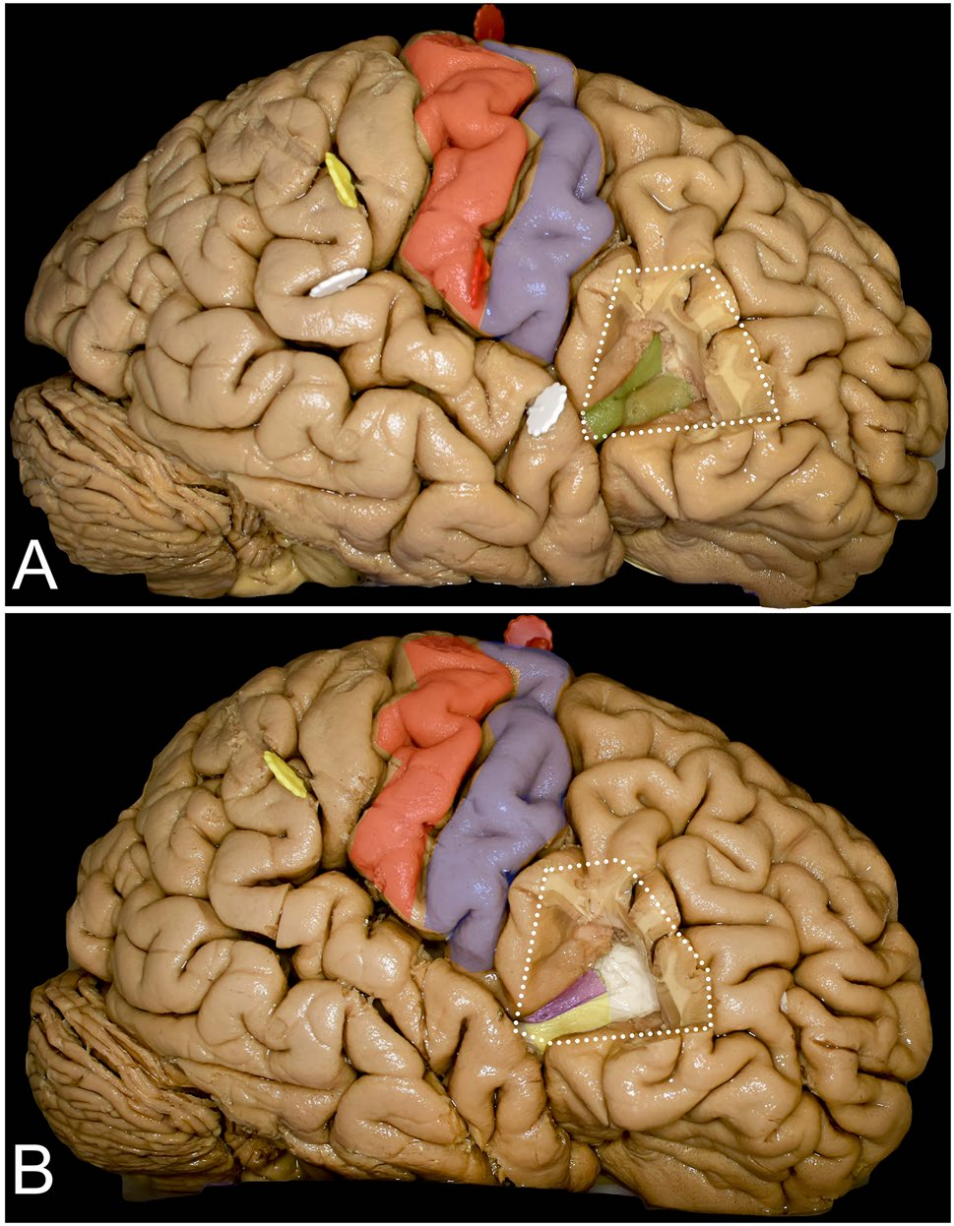
Exposure of the insular cortex and subcortical region was achieved with frontal operculum removal. **A** Resection of the pars triangularis (white dots) of the IFG provided exposure of the superior aspects of the anterior (light green) and anterior half of the middle short (darker green) gyrus. **B** On the subcortical level, this area corresponds to the frontal horn of the lateral ventricle (white) and below to the putamen (purple) posteriorly and the external capsule (yellow) anteriorly

Five adult brains were fixed with 4% formalin for at least 4 weeks. The brain was allowed to dry, placed on a tray inside a freezer which was at a temperature of − 15 °C and frozen for 2 weeks. For thawing and then for preservation, a 4% formalin solution at room temperature was used. Details of the technique were described previously [18]. The assessment of cortical anatomy was performed with the naked eye, whereas white matter dissection was performed with microsurgical tools and microscopic magnification in a stepwise manner. In the first step, cortical anatomy of the brain surface was assessed, and all measurements on the surface were performed. In the next step, frontal, temporal, and parietal opercula were sequentially resected en bloc to study and describe the surgical exposure achieved with each operculum. After performing measurements of the insula through each of the exposures at the cortical level, each operculum was restored in its original localization with a pin. In the next step, white matter dissection within the insula borders was performed to visualize the main subcortical structures from the neurosurgical point of view. After that, the removed opercula were once more restored, and the exposure of these subcortical structures through each of the opercula was analyzed. Anatomical correlations of the opercula to the insular landmark and to the subcortical white matter were assessed. The definition of each of the opercula used in the manuscript is presented in Figs. 1, 2, 3, 4, 5, and 7. The exposure achieved through each of the approaches was correlated with the Berger-Sanai classification of insular gliomas [45] (Fig. 2C). As the transcortical approach requires different view trajectories for each operculum in cortical- and subcortical-level photographic documentation, 6 photos were taken from different angles parallel to the insular surface, from anterior and posterior perspectives; each photo was taken with the lens directed superiorly and inferiorly. For presentation of the results, one perspective presented the widest view angle, which was chosen at both the cortical and subcortical levels. A digital camera (NikonD7200 with a Nikon DX 35 mm lens 1:1.8 G) was used for image documentation. Measurements were made with an electric digital caliper, protractor, and measuring tape. Color markers seen in figures correspond to anatomical landmarks and were placed for better anatomical orientation when the position of the brain or observation perspective was changed.

## Results

The presented data are a descriptive analysis of the surgical anatomy of the insula and related white matter tracts. Morphometric assessment was performed at each step of exposure.

### *Opercula (Table **1**)*

**Table 1:** Measurements related to the insular opercula

	Average	Range
Frontal operculum		
Horizontal ramus length	16.2 mm	1–27 mm
Horizontal ramus to the lateral sulcus	84.3°	13–150°
Ascending ramus	16.7 mm	11–21 mm
Ascending ramus to the lateral sulcus	105.7°	90–120°
Width of pars triangularis superiorly	19.5 mm	9–34 mm
Width of pars triangularis inferiorly	10.2 mm	5–23 mm
Parietal operculum		
Ascending ramus to IRP	16.8 mm	10–27 mm
Ascending ramus to postcentral sulcus	35.8 mm	30–42 mm
Central sulcus to the lateral sulcus	136.4°	121–156°
Postcentral sulcus to the lateral sulcus	119.3°	100–140°
Temporal operculum		
Temporal pole to sulcus acusticus (limited)	41.1 mm	30–52 mm
Temporal pole to posterior to Heschl’s gyri (extended)	35.1 mm	19–49 mm

Pars triangularis of the inferior frontal gyrus (IFG) constitutes the frontal entry point to the insula. The anterior border of the pars triangularis constitutes the horizontal ramus of the lateral sulcus, which is approximately 16.2 (range from 1 to 27) mm and is located at approximately 84.3 (range from 13 to 150) degrees in relation to the posterior ramus of the lateral sulcus. The posterior margin was marked by the ascending ramus of the lateral sulcus, which was 16.7 (range from 11 to 21) mm and 105.7 (range from 90 to 120) degrees to the posterior ramus of the lateral sulcus. The width of pars triangularis was approximately 19.5 (range from 9 to 34) mm superiorly and 10.2 (range from 5 to 23) mm inferiorly. The distal end of the horizontal ramus within the IFG was localized approximately 17.6 (range from 6 to 34) mm above the base of the frontal lobe.

The parietal operculum is constituted by the inferior segment of the postcentral gyrus, involving the primary sensory cortex. This region is located behind the inferior Rolandic point (IRP) and anterior to the postcentral sulcus. The IRP and postcentral sulcus are approximately 16.8 (range from 10 to 27) mm and 35.8 (range from 30 to 42) mm posterior to the ascending ramus of the lateral sulcus, respectively. The central sulcus is located at approximately 136.4 (range from 121 to 156) degrees, whereas the postcentral sulcus is located at approximately 119.3 (range from 100 to 140) degrees in relation to the posterior ramus of the lateral sulcus.

The temporal operculum is constituted by the superior temporal gyrus (STG). In the limited approach, resection ended at the sulcus acusticus, which was located approximately 41.1 (range from 30 to 52) mm from the temporal pole. When the extended approach was used, resection included Heschl’s gyri, and the length of the approach was extended by an additional 35.1 (range from 19 to 49) mm posteriorly.

### Insula (Figs. 3 and 4) (Table 2)

**Table 2:** Measurements related to the insular surface

Structures	Average	Range
Length of the anterior ramus of the limiting sulcus	28.3 mm	26–31 mm
Length of the superior ramus of the limiting sulcus	51.0 mm	50–53 mm
Length of the inferior ramus of the limiting sulcus	35.0 mm	28–39 mm
Length of the central sulcus of the insula	27.6 mm	25–33 mm
The central sulcus of the insula to the lateral sulcus	135°	125–140°
Length of the anterior long gyrus	31.6 mm	28–38 mm
Length of the posterior long gyrus	29.3 mm	28–31 mm
Length of the anterior short gyrus	24.7 mm	17–29 mm
Length of the middle short gyrus	24.0 mm	17–29 mm
Length of the posterior short gyrus	26.3 mm	21–29 mm
Depth from cortical surface to insular apex	18.0 mm	15–21 mm
Depth to limen insulae from the apex	17.0 mm	15–18 mm
Depth to the anterior insular point	24.3 mm	23–26 mm
Depth to the posterior insular point	35.0 mm	33–36 mm

The anterior ramus of the limiting sulcus is 28.3 (range from 26 to 31) mm, 51.0 (range from 50 to 53) mm superiorly, and 35.0 (range from 28 to 39) mm inferiorly. The central sulcus, which divides the short gyri anteriorly from the long gyri, is located 135 (range from 125 to 140) degrees to the lateral sulcus and has a length of approximately 27.6 (range from 25 to 33). The anterior and posterior long gyri are approximately 31.6 (range from 28 to 38) mm and 29.3 (range from 28 to 31) mm, respectively. The anterior, middle, and posterior short gyri are 24.7 (range from 17 to 29), 24.0 (range from 17 to 29), and 26.3 (range from 21 to 29) mm, respectively. The insular apex is located approximately 18.0 (range from 15 to 21) mm depth from the cortical surface, and the base of the limen insulae is located at an additional depth of 17.0 (range from 15 to 18) mm within the anterior sylvian point (ASP). The anterior insular point (AIP) is located at the level of the distal end of the horizontal ramus of the lateral sulcus at depths of 24.3 (range from 23 to 26) mm and 63.3 (range from 45 to 75) degrees in relation to the posterior ramus of the lateral sulcus. The posterior insular point (PIP) from the same pivot point is located at approximately 143.3 (range from 135 to 150) degrees at 35.0 (range from 33 to 36) mm in depth with respect to the cortical surface.

### *Frontal operculum (Fig. **5**) (Table **3**)*

**Table 3:** Exposure of the insula through the opercula

	Anterior limiting sulcus	Superior limiting sulcus	Inferior limiting sulcus
Frontal operculum	17.7 mm (range from 14 to 20) mm	15.7 mm (range from 15 to 18) mm	––––––––––––
Parietal operculum	––––––––––––	11.7 mm (range from 8 to 17) mm	––––––––––––
Temporal operculum, limited	11.3 mm (range from 9 to 14) mm	––––––––––––	3.5 mm (range from 0 to 10) mm
Temporal operculum, extended	11.3 mm (range from 9 to 14) mm	––––––––––––	35.0 mm (range from 28 to 39) mm

Removal of the pars triangularis provided exposure of the AIP in all cases at the anterior–superior limit of the exposure, approximately at the level of the distal end of the horizontal ramus of the lateral sulcus. The superior upper half of the anterior limiting sulcus (mean 17.7, range from 14.0 to 20.0 mm) and the anterior one-third (mean 15.7, range from 15.0 to 18.0 mm) of the superior limiting sulcus up to the midpoint of the middle short gyrus were exposed. The tip of the ascending ramus of the lateral sulcus points just anterior to the sulcus between the anterior and middle short gyrus. The line connecting the tip of the horizontal and ascending sulci follows the anterior segment of the superior insular sulcus and is almost parallel to the inferior frontal sulcus. Most of the posterior short and posterior half of the middle short gyri are covered by the pars opercularis of the IFG and the inferior aspect of the precentral gyrus. The central sulcus of the insula is identified just under the central sulcus of the hemisphere and is directed anteriorly. Most of the central sulcus is covered by the inferior segment of the precentral gyrus. The anterior half of the anterior limiting sulcus is covered by the pars orbitalis of the IFG, whereas the inferior limiting sulcus and the limen insulae are covered by the STG. At the subcortical level, the mesial limit of resection through the frontal operculum is limited superiorly and anteriorly by the frontal horn of the lateral ventricle. Below the lateral ventricle, under the external capsule formed by the uncinate fascicle/inferior frontoocipital fascicle (UF/IFOF) complex, the putamen and head of the caudate are visible. Notably, this approach provides access mainly to zone I.

### *Parietal operculum (Fig. **6**) (Table **3**)*

**Fig. 6:**
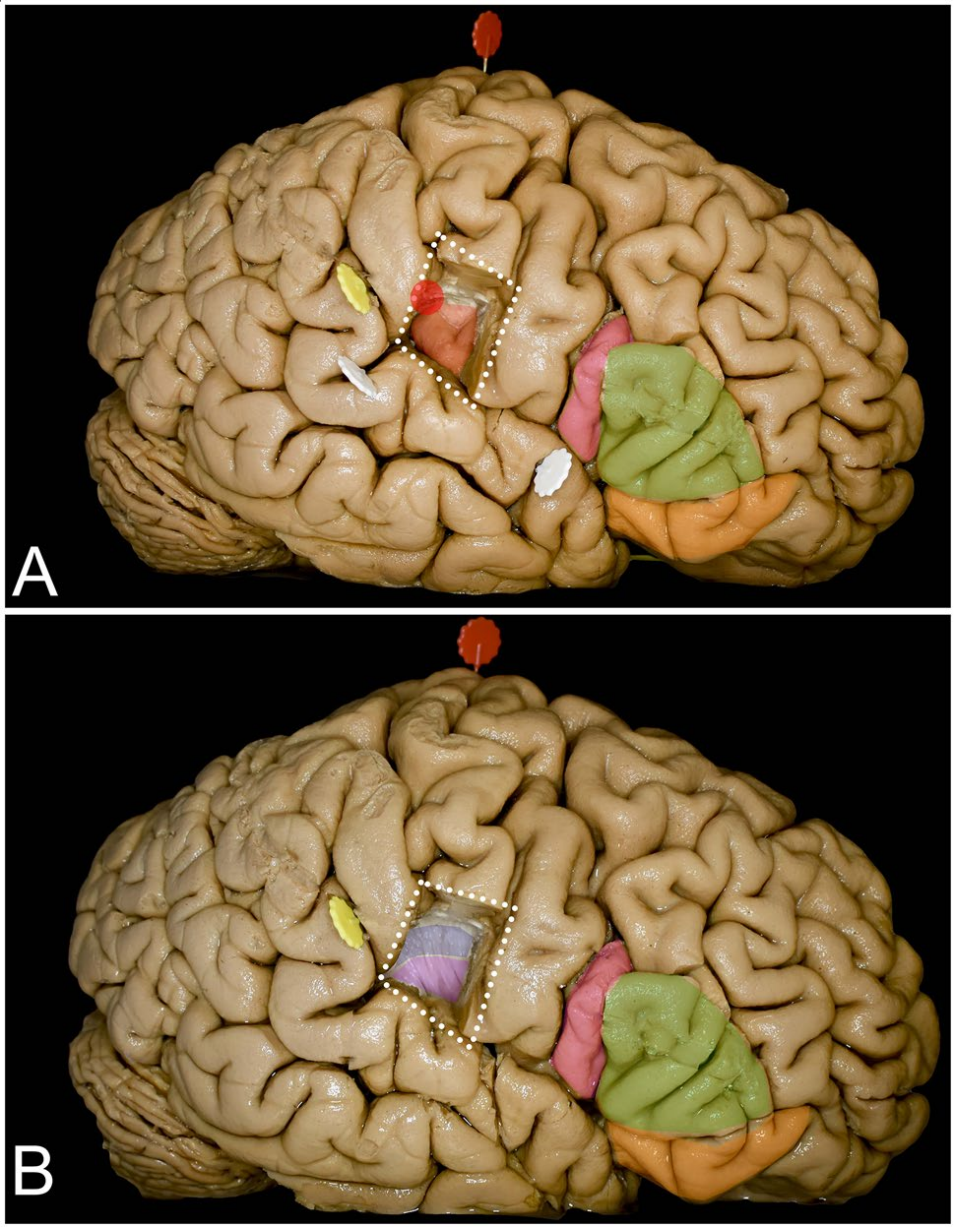
Exposure of the insular cortex and subcortical region was achieved with parietal operculum removal. A Resection of the inferior aspect of the postcentral gyrus (white dots) provided exposure of the superior-posterior compartment of the insula, including the PIP (red dot). The superior half of the anterior long gyrus (light red) is also exposed, whereas the central sulcus of the insula is covered by the precentral gyrus of the hemisphere and the posterior long gyrus (dark red) by the STG. B On the subcortical level, just under the superior limiting sulcus, the corona radiata (blue) corresponding to the corticospinal tract, not covered by the putamen (purple), is exposed. Below that, the putamen (purple), which covers medially the internal capsule, is identified

The inferior segment of the postcentral gyrus was removed to expose the posterior one-fifth of the superior limiting sulcus (mean 11.7, range from 8 to 17 mm) and expose the PIP. The line connecting the distal end of the anterior ascending sulcus and the lowest point of the postcentral sulcus runs approximately along the middle and posterior segments of the superior limiting sulcus. The IRP marks the central sulcus of the insula between the posterior short and anterior long gyrus and is not visualized with resection of the parietal operculum without retraction of the precentral gyrus. The superior half of the anterior long gyrus (mean 14.7, range from 13.0 to 17.0 mm) is exposed as well as the posterior long gyrus. Resection of the parietal operculum provided exposure of the superior aspect of Heschl’s gyri within the temporal operculum, and when following them medially and slightly superiorly, the PIP could be identified. At the subcortical level, the arcuate fascicle/superior longitudinal fascicle (AF/SLF) complex can be encountered when corticotomy is performed high above the lateral sulcus. To visualize the superior limiting sulcus, 17.2 (range from 13.0 to 22.0) mm of the postcentral gyrus must be resected. In the superior region of the exposed insula under the cortex short segment (mean 9.3 mm, range from 7 to 10 mm) of the corona radiata corresponding to the corticospinal tract above the putamen, the superior limiting sulcus is covered just by the external capsule, providing the mesial border of resection and is located at approximately 38.0 mm (range from 30 to 40 mm). The posterior limb of the internal capsule is covered in whole by the putamen, which indicates the mesial resection border under the corona radiata. This approach provides limited exposure of zone II, especially in its superior-posterior aspect.

### Temporal operculum (Fig. 7) (Table 3)

When resection of the temporal operculum is limited up to the sulcus acusticus, a short anterior segment of the inferior limiting sulcus (mean 3.5, range from 0 to 10.0 mm), limen insulae and inferior one-third of the anterior limiting sulcus (mean 11.3, range from 9 to 14.0 mm) are exposed. Exposure of the inferior segments of all short gyri with confluence within the apex and of the anterior long gyrus is achieved. Providing an extended approach by adding resection of the posterior aspect of the STG with Heschl’s gyri, the whole length of the inferior limiting sulcus, posterior long gyrus, and anterior long gyrus except the short superior segment just behind the central sulcus of the insulae are exposed. The line connecting the distal end of the lateral sulcus with the sulcus acusticus approximately corresponds to the position of the inferior limiting sulcus. Extension of the STG resection has no added value in terms of the exposure of the anterior part of the insulae. At the anteroinferior portion of the insula, at the level of the limen insula, the UF/IFOF complex is identified at approximately 28.0 (range from 27.0 to 29.0) mm from the cortical surface. Both tracts are most compacted there and spread apart posteriorly and anteriorly at this point. Mesial to the UF/IFOF complex, the anterior perforated substance with perforators from the MCA entering through the anterior perforated substance is exposed. The most lateral part of the anterior perforated substance is located approximately 9.5 (range from 9.0 to 10.0) mm from the superficial fibers of the UF/IFOF complex. Under the long gyri, posterior to the central sulcus, the mesial border is set by the putamen except for the short segment just under the superior limiting sulcus, where the uncovered corona radiata is reached. This approach provides exposure mainly to zones III and IV.

## Discussion

In the literature, a shift from the original TS to the TC approach for resection of insular gliomas, especially in larger volume tumors with significant posterior extension, has recently been observed [3, 8, 30, 39, 48].

### Rationale behind transopercular approach

According to a recent literature review, the rates of complete resection are not affected by the approach used during the procedure. However, the risk of permanent deficits related to surgical procedures within the insula is much lower in patients undergoing an operation through the TC approach, despite the higher rate of immediate postoperative deficits [8]. Postoperative morbidity in insular gliomas is related to vascular eloquent cortex within opercula or white matter tract injury. Immediate postoperative deficits are explained by resection-induced contusion, edema, and hypoperfusion, whereas permanent deficits are related mainly to infarctions related to MCA branches on the lateral surface of the insula and the lenticulostriate arteries (LSAs) within the anterior perforated substance on its mesial side [7]. Postoperative ischaemia is observed in approximately one-quarter of patients undergoing the TC or TS approach within the whole insula, despite the tumors within zone II, where it reaches almost half of the patients exclusively in the TS group [39]. This result is related to the limited exposure of the posterior zones (zones II and III) of the insula to the TS due to arterial and venous anatomical limitations [25]. In addition to language function, awake conditions in both the dominant and nondominant hemispheres have a role in the preservation of functions such as movement, vision, or high cognitive function at the cortical and subcortical levels. In terms of movement, only awake conditions allow us to identify regions related to visuospatial processing or spatial cognition, which is of greatest importance in daily activities. The benefit of mapping in awake conditions is also apparent for the avoidance of hemianopsia or to preserve functions such as calculation, memory or emotion.

### Preoperative assessment and selection of the approach

In preoperative planning, in addition to structural MRI, functional MRI (fMRI) and diffusion tensor imaging (DTI) tractography can provide a three-dimensional anatomical perspective of the tumor and surrounding eloquent cortex and white matter tracts. On the cortical level, despite establishing the dominant hemisphere in terms of language function with the use of fMRI, identification of motor-, sensory-, or visual-related cortices within the frontal, parietal, and occipital lobes can be achieved. The relationship of the tumor to white matter tracts can be established with the use of DTI tractography. Despite technical advancements, both fMRI and DTI still present some drawbacks that make direct brain stimulation the gold standard in the intraoperative identification of eloquent regions at the cortical and subcortical levels. The limitations of MRI techniques are related to parenchymal invasion, pathological angiogenesis, and disturbed neurotransmitter concentrations due to glioma invasion [21, 40]. Intraoperative usage of preoperative imaging with neuronavigation is additionally limited by the observed brain shifts from tumor debulking and cerebrospinal fluid loss. Intraoperatively, neuronavigation coupled with intraoperative ultrasound with the ability to correct brain shifts can be applied to optimize the use of neuroimaging and simultaneously make resections safer [26]. In the case of low-grade gliomas, intraoperative MRI is very useful to confirm the extent of tumor removal at the end of resection to avoid leaving some tumor behind in noneloquent regions [6]. Intraoperative brain mapping has no side effects from any of the abovementioned techniques. As long as the surgeon is aware of the localization and function of the white matter tracts and their three-dimensional relationship to the tumor, function-based limited resection can be applied.

**Fig. 7:**
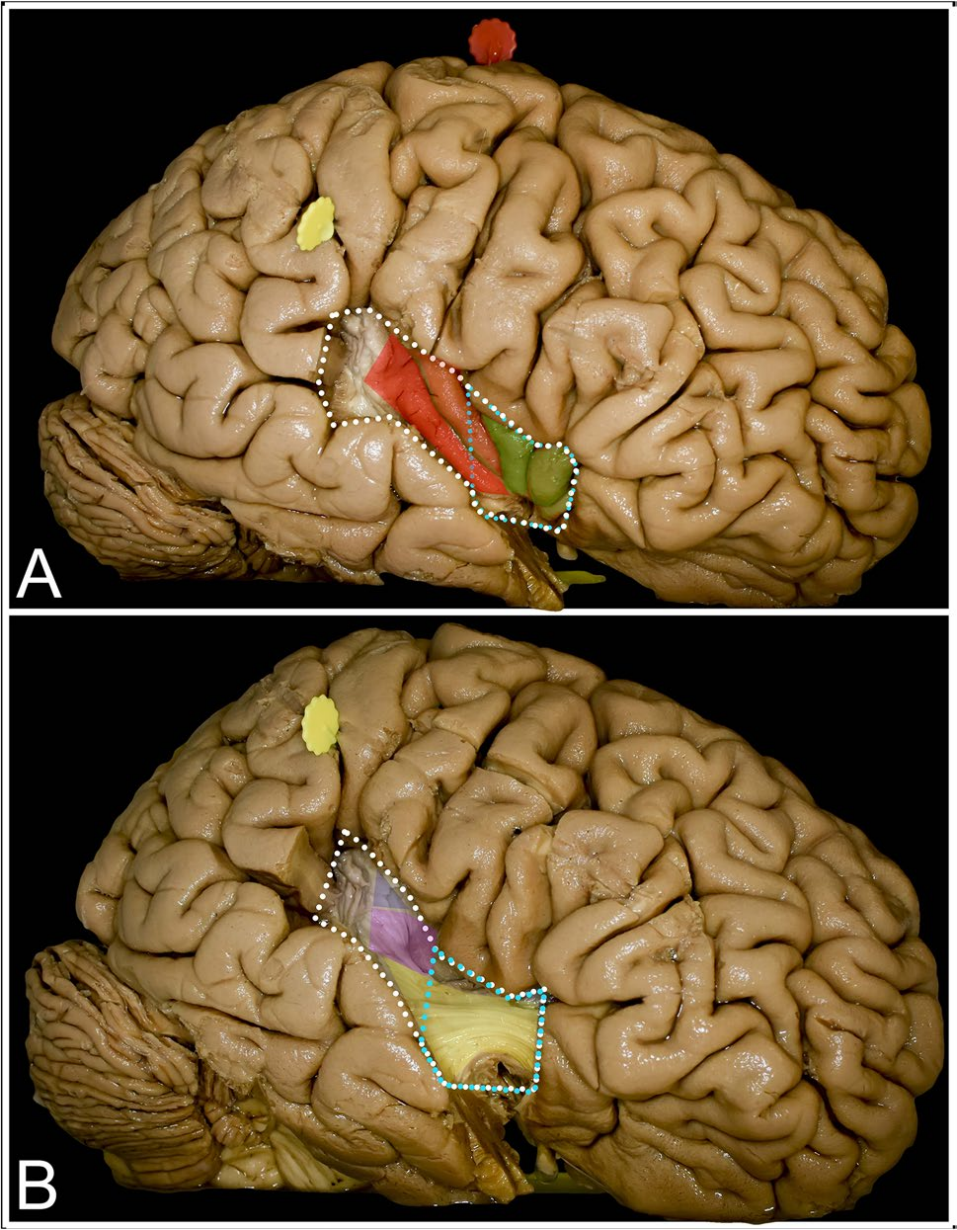
Exposure of the insular cortex and subcortical region was achieved with limited (light blue dots) and extended (white dots) temporal operculum removal. **A** Resection of the anterior segment of the STG provided exposure of the inferior aspect of the anterior liming sulcus, the limen insulae and a very limited segment of the inferior limiting sulcus. The inferior segments of the short gyri (green), including the insular apex, and of the anterior long gyrus (light red) were exposed. By extending the approach by resecting Heschl’s gyri, the whole length of the inferior limiting sulcus, of the posterior long gyrus (dark red) and of almost the whole length of the anterior long gyrus except the short superior segment was visualized. **B** With limited resection, mainly the UF/IFOF complex (yellow) is exposed within the limen insulae, which indicates the mesial border of resection. Posteriorly, the putamen (purple) covers the posterior limb of the internal capsule states for the medial border of resection. At the superior-posterior aspect of the exposure, the corona radiata (blue) is identified

Despite the described Sanai and Berger classification of insular gliomas, other based on anatomical localization (Yasargil) or on extension within white matter tracts (Mandonnet and Duffau) are in use [31]. Based on the location of the tumor within the insula, a single frontal, temporal, or parietal approach or different combinations of opercular routes can be chosen to access the tumor. Tumors located within the anterior–superior aspect of the insula (zone I) (Fig. 5) can be reached through the frontal operculum and those within the anterior-inferior aspect of the insula (zone IV) (Fig. 7) can be accessed with the limited temporal approach. Extended temporal approach provides exposure of the whole insula located below the lateral sulcus (zones III and IV) (Fig. 7) and to the inferior aspect of zone II. Tumors within zone II (Fig. 6) within the posterior-superior segment of the insula state for tumors that carry the highest risk of permanent neurological deficit in TS and TC approaches, mainly due to vascular incidents [25, 34]. Despite the localization and size of the tumor, it is advisable that the craniotomy provides visualization of all opercula; details are presented in Fig. 2 [27]. In terms of awake craniotomy despite tumor localization, it is beneficial to expose the central sulcus to utilize the lowest and safest approach in terms of the risk of intraoperative seizure parameters for direct cortical and subcortical stimulation. Stimulation of the precentral gyrus results in positive responses on the lowest thresholds, and stimulation parameters are reproducible at the subcortical level [4, 17]. Having parameters of the stimulation set, identification of the eloquent cortex within the frontal, temporal, and parietal opercula can begin.

### The trans-frontal operculum approach

**Fig. 8:**
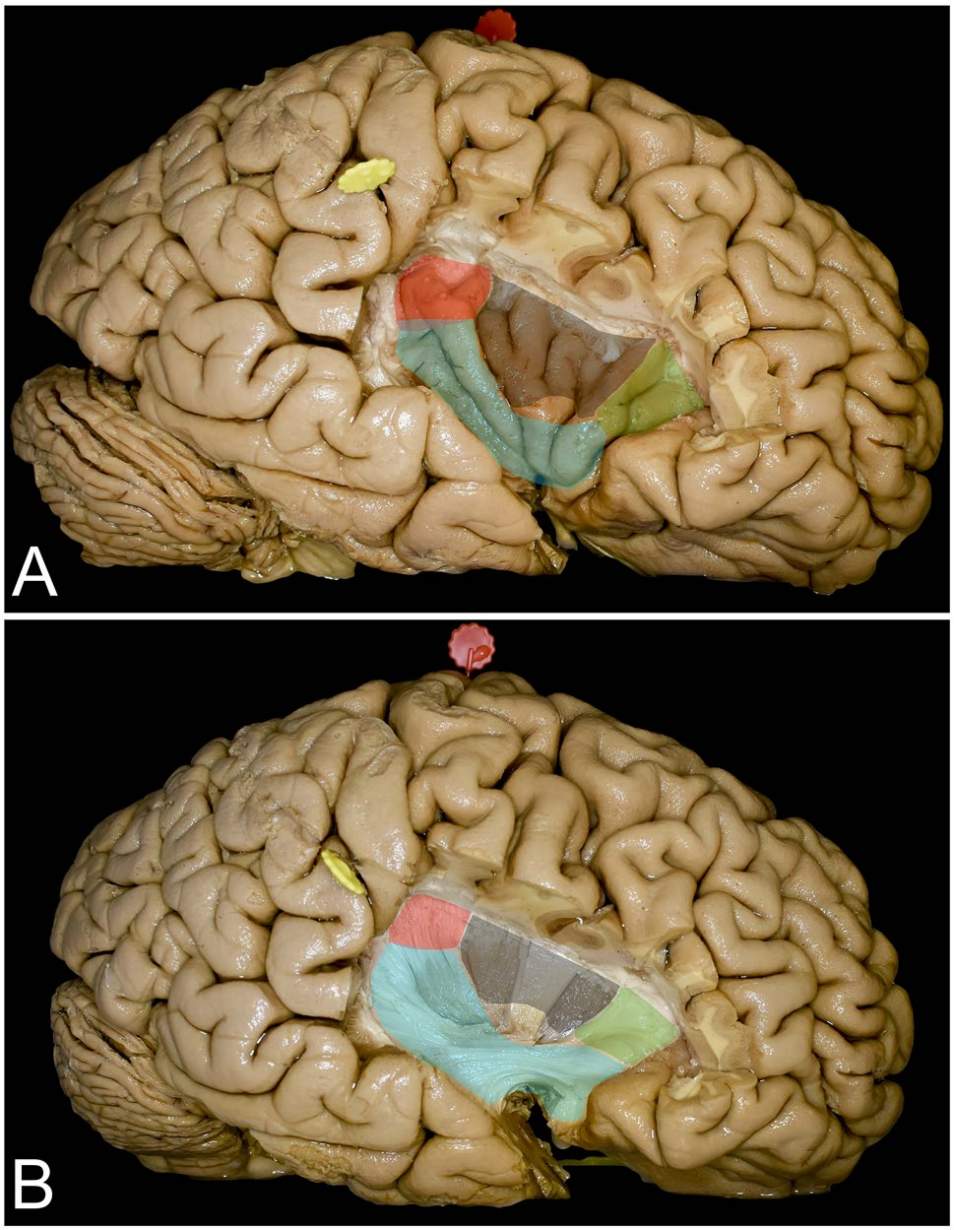
Exposure of the insular cortex and subcortical region shown following removal of all opercula. **A** The figure presents the exposure of the insular cortex through the frontal (green), parietal (red), and temporal (light blue) opercula. The region marked in black corresponds to the region of the insula that cannot be exposed without retraction of the pars opercularis of the IFG and the precentral gyrus. The noncolored region corresponds to the insular apex that is visualized mostly within the anterior sylvian point without any opercular retraction or resection. **B** The figure presents the exposure of subcortical structures that indicate the mesial border of resection through different opercula. The colors used here represent the same opercula as in Fig. 8A

The frontal operculum is constituted by the pars orbitalis, triangularis, and opercularis of the IFG (Figs. 1, 2, and 5). Within the frontal lobe, only the posterior part of the IFG is related to speech production [42]. The key region to identify is the pars opercularis of the IFG, which provides a landmark for the ventral premotor cortex, whereas the pars triangularis is most often not involved in the so-called Broca’s area; its resection provides exposure of zone I of the insula (Figs. 5 and 8) [12, 54]. On the dominant side, intraoperative stimulation of the frontal operculum results in arrest of speech and motor function, whereas on the nondominant side, intraoperative stimulation of the frontal operculum only arrests motor function on the contralateral side. For intraoperative mapping within the frontal operculum, simple counting from 1 to 10 can be performed to evaluate language function and finger tapping can be used to evaluate motor function to assess the arrest of speech and movement. From a practical point of view, it is crucial to distinguish anomia from speech arrest. For this reason, the patient is asked to answer by providing a full sentence, such as “This is a…”, and finally name the visualized object [17]. Following resection of the superficial cortical part of the insula, the same parameters of stimulation are retained for active mapping at the subcortical level to identify mesial borders of resection. The frontal horn of the lateral ventricle, which is located just under the superior limiting sulcus and AF/SLF complex (Figs. 1 and 5b), serves as an anatomical landmark. Intraoperatively below the lateral ventricle, the putamen indicates the defined mesial border of resection, which can result in difficulties in articulation when stimulated regardless of the side undergoing operation [11, 34]. Below, closer to the level of the insular apex and the limen insulae, the anterior-inferior deep limit of resection can be identified by actively mapping the UF/IFOF complex. Stimulation in the dominant hemisphere during language tasks produces semantic paraphasia, and stimulation in the nondominant hemisphere during semantic association tasks creates nonverbal semantic disorders [14, 16, 28]. Insular gliomas seem to grow in the space between the UF/IFOF complex and insular convexity, displacing and compressing the UF/IFOF complex medially [33]. This space is very narrow in nontumoral hemispheres and is approximately 3 mm, whereas in tumoral hemispheres, it can extend up to 21 mm [33]. Identification of the UF/IFOF complex is important from a surgical point of view in terms of the risk of injury to LSAs related to anterior perforating substances [20, 29, 32, 55]. As these small branches provide vascular supply to the basal ganglia and internal capsule, it is reasonable to leave a small amount of the tumor medially to the UF/IFOF complex at the level of the temporal stem to avoid injuring them [9]. The deficit related to UF injury, even in the dominant hemisphere, can be compensated and is not associated with permanent deficits [15]. Injury to the IFOF on either side results in permanent cognitive dysfunction regarding semantic processing [35]. Within the central and posterior-inferior regions, the putamen represents the deep landmark for resection and covers the posterior limb of the internal capsule. Stimulation of the putamen regardless of the operated side results in articulation difficulties [11].

### The trans-parietal operculum approach

Resection of the parietal operculum is required to reach the superior-posterior region of the insula within zone II (Figs. 6 and 8). The superior-posterior region of the insula is covered by the inferior segment of the postcentral gyrus, which, when stimulated, results in different sensations mainly within the face and mouth. Additionally, this approach carries a risk of going deep enough to involve the AF/SLF complex, which is located just above the superior limiting sulcus. In the dominant hemisphere, intraoperative stimulation may result in language processing disturbances such as articulation (also observed in the nondominant hemisphere), phonological processing, and repetition [10] (Fig. 1). In the nondominant hemisphere, stimulation of the AF/SLF complex may induce spatial neglect symptoms or vertigo, especially when stimulated within the temporoparietal junction [2, 49]. Most commonly, this operating window provides visualization of the PIP. This region is located approximately 1 cm deeper than the AIP, which makes approaching this region even more challenging. Resection of tumors within zone II entails the highest risk for direct injury of the posterior limb of the internal capsule, resulting in motor deficits [13, 24, 29]. Within this zone, there is a narrow segment of the corona radiata just under the superior ramus of the limiting sulcus, which is not covered by the putamen. Here, they enter the internal capsule, which forms the medial limit of resection. Stimulation of the corona radiata may result in responses in terms of muscle contraction within the face and extremities. The most lateral fibers within the corona radiata are responsible for the sensorial aspect of the movement and the state of the thalamocortical pathway. Stimulation of these fibers may result in clumsy movements; hence, it would be beneficial to map the internal capsule in awake conditions [9].

### The trans-temporal operculum approach

The object-naming test is widely used to identify the posterior border of resection within the temporal lobe [38]. Positive stimulations result in anomia or phonemic paraphasia have been observed in the dominant hemisphere [19] On the nondominant side, resection can be extended beyond Heschl’s gyri, which can result in auditory agnosia; long-term follow-up in most patients did not reveal in permanent deficits [44, 45]. Resection of the STG results in the widest exposure of the insula, mainly of the inferior compartments (zones III and IV) of the insula (Figs. 7 and 8). Despite identification of the AF/SLF complex at the posterior limit of the resection and of the UF/IFOF complex within the temporal stem, the trans-temporal operculum approach is related to visual function, as optic radiation can be identified over the lateral wall of the temporal horn of the lateral ventricle. Intraoperative visual field testing during direct brain stimulation may result in blurred vision, phosphenes, hallucinations, or shadows [23]. The aim of intraoperative mapping is to avoid hemianopsia.

### Blind spots of the transcortical approach

Combining all approaches, exposure of the whole insula except the region anterior to the central sulcus of the insula, mainly the short gyrus under the precentral gyrus of the hemisphere, is achieved (Fig. 8). Resection of the inferior part of the precentral gyrus may result in central facial paralysis, which may cause permanent articulation deficits in some cases and should not be removed [5, 13]. The covered regions of the insula can be reached with minimal retraction of the preserved brain. When surgery is performed in an asleep-awake-asleep protocol, the orbitofrontal and temporopolar regions of the tumor can be removed in asleep conditions at the end of surgery. This might be beneficial due to the painful sensations that are sometimes observed related to dura manipulations when the tumor is resected in proximity to the skull base.

### The function of the insula

Even though the insular cortex constitutes a negligible percentage of the cortical surface of the brain, it is related to approximately 20 different functions and pathological states [37]. The concept of the anterior insula with pyramidal cells (dysgranular area) and the posterior insula with granule cells separated at the level of the central sulcus of the insula is related not only to histological studies but also to function. The function is related to interconnections with different parts of the brain. The anterior insula contains multiple functions such as taste, olfaction, visceromotor control (including cardiac rate and rhythm control), somatomotor control with abilities in motor function recovery, speech production, cognitive control, body awareness, self-recognition, and individual and social emotions. The anterior insula has a greater number of connections to the frontal cortex [37, 43, 46, 47, 56]. The posterior insula with auditory, vestibular, somatosensory, pain, and temperature or viscerosensation function has a greater number of connections with the cingulate and parietal cortices [1, 50, 56]. Changes in the insular cortex based on neuroimaging were identified in patients suffering from schizophrenia, conduct disorder, frontotemporal dementia, or drug addiction [36, 37, 51, 52].

### Limitations of the study

In the transcortical approach, the exposure of the insula is limited by intraoperative mapping, which is not possible to assess in the preoperative period or in anatomical studies. Additionally, the physical parameters of cadaveric brain tissue may not exactly present an intraoperative brain structure. This study showed that the optimal surgical approach for insular gliomas requires assessment of tumor localization within the insula, which may be easier when orienting based on constant cortical and subcortical anatomical landmarks. Due to the limited number of specimens used in the study, readers should carefully consider our results, as we were not able to describe all possible variants of the anatomy. Additionally, the anatomical relationship between the structures in patients with tumors is disturbed by the tumor mass or during tumor removal due to brain shifts related to cerebrospinal fluid loss and tumor debulking. A better surgical perspective of the operated field could be achieved with specimens with injected arterial and venous system vessels. Despite the abovementioned constraints, knowledge of normal anatomy is of greatest importance even when the anatomy is disturbed by a tumor.

### Summary of the findings

Proper anatomical definition of a tumor within the insula is crucial for proper planning of the approach through the opercula. Some regions of the insula can be reached exclusively by each operculum, such as the AIP through the frontal operculum, and the PIP through the parietal operculum, and the limen insulae through the temporal operculum. Anatomical three-dimensional visualization of the main tracts within the insula, such as the UF/IFOF complex around the limen insula, corona radiata, and internal capsule within the posterior compartment, and the AF/SLF complex on the superior and posterior borders of the insula, allow proper selection of intraoperative tests for brain mapping during direct electrical stimulation during awake procedures. The region of the limen insulae is critical in terms of the risk of vascular injury related to the lenticulostriate arteries, which are directly covered by the UF/IFOF complex. The superior-posterior region of the insula is critical in terms of direct injury to the corticospinal tract where the corona radiata is not covered by the putamen.

## Data Availability

The authors confirm that the data supporting the findings of this study are available within the article.
